# Vitamin D Deficiency During Pregnancy Is Associated With Greater LDL‐C Increase, Elevated β‐Hydroxybutyrate and Altered Neonatal Metabolic Markers—A Secondary, Pooled Analysis of the Randomized, Controlled Vitamin D and Lifestyle for Gestational Diabetes Prevention Trial (DALI)

**DOI:** 10.1002/edm2.70258

**Published:** 2026-08-13

**Authors:** Jürgen Harreiter, Teresa Gisinger, Lilian C. Mendoza, David Simmons, Gernot Desoye, Roland Devlieger, Sander Galjaard, Peter Damm, Elisabeth R. Mathiesen, Dorte M. Jensen, Lise Lotte T. Andersen, Fidelma Dunne, Annunziata Lapolla, Maria G. Dalfra, Alessandra Bertolotto, Ewa Wender‐Ozegowska, Agnieszka Zawiejska, David Hill, Judith G. M. Jelsma, Frank J. Snoek, Christof Worda, Dagmar Bancher‐Todesca, Mireille N. M. van Poppel, Rosa Corcoy, Alexandra Kautzky‐Willer

**Affiliations:** ^1^ Division of Endocrinology and Metabolism, Department of Medicine III Medical University of Vienna Vienna Austria; ^2^ Department of Medicine Landesklinikum Scheibbs Scheibbs Austria; ^3^ Institut D'investigació Biomèdica Sant Pau (IIB SANT PAU) Barcelona Spain; ^4^ CIBER‐BBN, ISCIII Madrid Spain; ^5^ Macarthur Clinical School Western Sydney University Sydney New South Wales Australia; ^6^ Department of Obstetrics and Gynecology Medical University of Graz Graz Austria; ^7^ Department of Development and Regeneration KU Leuven, University Leuven Leuven Belgium; ^8^ Department of Obstetrics and Gynecology University Hospitals Leuven Leuven Belgium; ^9^ Department of Obstetrics Gynecology and Fertility, GZA Sint‐Augustinus Wilrijk Belgium; ^10^ Department of Obstetrics and Gynaecology, Division of Obstetrics and Prenatal Medicine, Erasmus MC University Medical Centre Rotterdam Rotterdam the Netherlands; ^11^ Departments of Endocrinology and Obstetrics, Rigshospitalet, Department of Clinical Medicine, Faculty of Health and Medical Sciences, Center for Pregnant Women With Diabetes University of Copenhagen Copenhagen Denmark; ^12^ Steno Diabetes Center Odense Odense University Hospital Odense Denmark; ^13^ Department of Gynaecology and Obstetrics, Odense University Hospital, Odense, Denmark & Department of Clinical Research, Faculty of Health Sciences University of Southern Denmark Odense Denmark; ^14^ Institute for Clinical Trials University of Galway Galway Ireland; ^15^ Universita Degli Studi di Padova Padova Italy; ^16^ Azienda Ospedaliero‐Universitaria Pisana Pisa Italy; ^17^ Department of Reproduction, Chair of Feto‐Maternal Medicine Poznan University of Medical Sciences Poznan Poland; ^18^ Department of Medical Simulation, Chair of Medical Education Poznan University of Medical Sciences Poznan Poland; ^19^ Lawson Health Research Institute London Ontario Canada; ^20^ Amsterdam UMC, Vrije Universiteit Amsterdam, Department of Public and Occupational Health Amsterdam Public Health Research Institute Amsterdam the Netherlands; ^21^ Amsterdam UMC, Vrije Universiteit Amsterdam, Department of Medical Psychology Amsterdam Public Health Research Institute Amsterdam the Netherlands; ^22^ Division of Obstetrics and Feto‐Maternal Medicine, Department of Obstetrics and Gynecology Medical University of Vienna Vienna Austria; ^23^ Department of Human Movement Science, Sport and Health University of Graz Graz Austria; ^24^ Departament de Medicina Universitat Autònoma de Barcelona Barcelona Spain

**Keywords:** 25‐OH‐D3, birth outcome, cord blood, LDL‐C, lipids, pregnancy, pregnancy outcome, vitamin D, vitamin D deficiency, ß‐OH‐butyrate

## Abstract

**Introduction:**

Vitamin D (vitD) plays a role in metabolic regulation, including lipid metabolism and insulin sensitivity. During pregnancy, profound physiological changes in lipid handling and ketogenesis occur to support fetal development. However, the extent to which maternal vitamin D status influences these metabolic adaptations and fetal metabolic markers remains unclear.

**Methods:**

In this secondary analysis, we examined lipid distribution throughout pregnancy—from before 20 weeks' gestation to delivery—in women with overweight or obesity, stratified by vitamin D status (deficiency, insufficiency, or sufficiency), assessing both maternal and cord blood. Main inclusion criteria were: age > =18 years, singleton pregnancy, < 20 weeks' gestation, BMI ≥ 29 kg/m^2^. Women with GDM < 20 weeks' gestation were excluded. In total, 962 pregnant women were divided into vitD deficient (< 30 nmol/L, *n* = 102), insufficient (30–50 nmol/L, *n* = 222) and sufficient (> 50 nmol/L, *n* = 638) groups. VitD levels and lipid concentrations were assessed at < 20, 24–28 and 35–37 weeks' gestation and in cord blood.

**Results:**

Compared with vitD sufficient women, women with vitD deficiency had significantly larger increases in LDL‐C throughout pregnancy and ß‐OH‐butyrate at 24–28 weeks' gestation, in adjusted analysis. VitD in cord blood was highest in offspring of mothers with vitD sufficiency. In cord blood, significantly higher ß‐OH‐butyrate was observed with vitD deficiency; lipid concentrations were similar between groups.

**Conclusions:**

Early vitamin D deficiency before 20 weeks of gestation was associated with altered metabolic trajectories during pregnancy, including greater increases in LDL cholesterol and ketone body concentrations in women with overweight or obesity, as well as higher cord blood ketone levels in their offspring. These findings suggest that early maternal vitamin D status may influence maternal and fetal metabolic adaptations, although causal relationships and clinical implications require further investigation.

**Trial Registration:**

Trial registered at ISRCTN registry (https://doi.org/10.1186/ISRCTN70595832) trial number ISRCTN70595832. Registration date 02/12/2011

## Graphical abstract

Early pregnancy vitamin D deficiency in women with overweight or obesity is associated with greater increases in LDL cholesterol and ketone bodies during gestation, as well as higher cord blood ketone levels in offspring. These findings suggest a role of maternal vitamin D status in shaping metabolic adaptations in pregnancy and fetal exposure.

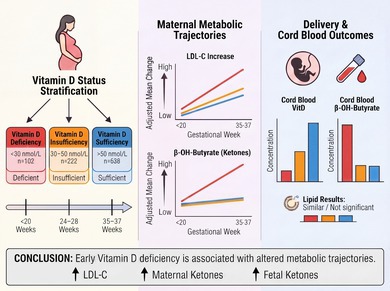

## 1 Introduction

The fat‐soluble vitamin D is a prohormone with endocrine, autocrine and paracrine functions predominately known for maintaining calcium balance and promoting bone health [1]. Several studies have shown the multifaceted impact of vitamin D on extra‐skeletal functions such as metabolism, inflammation, immune response, neuromuscular function, cell differentiation and proliferation, or reproductive physiology due to the distribution of vitamin D receptors across various organs and tissues throughout the body [1, 2, 3].

Several international societies define 25‐hydroxy‐vitamin D as vitamin D levels < 30 nmol/L (< 12 ng/mL), insufficiency between 30–50 nmol/L (12–20 ng/mL) and sufficiency above 50 nmol/L (> 20 ng/mL) [4]. Vitamin D deficiency poses a global health challenge affecting over one billion children and adults across the world and several patient groups such as pregnant women or patients with obesity are especially at high risk [5]. In Europe, the prevalence of vitamin D deficiency was estimated at 13% irrespective of age groups, latitude or ethnicity [6]. Vitamin D deficiency or insufficiency in pregnancy ranges from 5% to 84% worldwide with substantial regional differences [7] and an increased risk was observed in obese pregnant women with two‐fold higher numbers of inadequate maternal and neonatal vitamin D levels [8].

Vitamin D deficiency during pregnancy is associated with an elevated risk of adverse outcomes for both the mother and the offspring [9]. These risks include gestational diabetes, dyslipidemia, preeclampsia, preterm birth, low birth weight, obstructed labour, increased infection rates, and even potential impacts on fetal brain development and neonatal respiratory function [9]. However, inadequate maternal vitamin D concentrations might also be significant in offspring childhood and adolescence. Maternal vitamin D seems to be a relevant factor for their neural, musculoskeletal, psychomotor growth and bone health, with low maternal concentrations associated with higher risk for metabolic, psychiatric, neurologic, pulmonary or oncologic disease, as well as disturbances in skeletal development [10].

In early pregnancy, inadequate vitamin D concentrations have been associated with metabolic disease such as hyperglycemia or dyslipidemia [11, 12] but evidence is scarce and associations with cord blood analytes not sufficiently investigated. A nested case‐cohort study found that women in week 16 of pregnancy had a two‐fold higher gestational diabetes (GDM) risk in the lowest vitamin D quartile compared with the highest quartile [11]. A prospective cohort study observed that women with inadequate vitamin D concentrations defined as below 75 nmol/L had higher LDL cholesterol at baseline but also had increased triglyceride and LDL cholesterol changes later in pregnancy [12].

A small study revealed that even after an Institute of Medicine (IOM) recommended daily supplementation with 600 IU vitamin D throughout pregnancy, most pregnant women and their offspring were vitamin D deficient [13]. Recently we reported that vitamin D supplementation with 1600 IU vitamin D3 per day in overweight/obese pregnant women was able to sufficiently raise vitamin D3 levels throughout pregnancy in most overweight/obese pregnant women [14, 15]. However, this supplementation with vitamin D3 did not improve glucometabolic measurements in a clinically meaningful way and did not show any significant differences in maternal or neonatal lipid measurements and lipid distribution compared with the control group [14, 15].

Hormonal changes in pregnancy lead to increased triglyceride and cholesterol levels. However an excessive rise of triglyceride and cholesterol values are related to various adverse pregnancy outcomes such as preeclampsia, gestational diabetes, pancreatitis, preterm birth and fetal distress [16].

Ketone bodies—as 3‐hydroxy butyrate—can serve as an alternative energy source during pregnancy, especially in fasting conditions, conditions with hypoglycemia and in the third trimester of pregnancy as maternal insulin resistance increases and fetal energy demands grow [17]. Muscles, the heart and the brain can use ketone bodies as an energy resource. They may support fetal brain development, but high levels were linked to adverse fetal outcomes [17]. The conversion of vitamin D into its active form is prevented by an acidic environment derived from increased ketone bodies that inactivate hepatic and renal hydroxylases [18]. Furthermore, vitamin D binding proteins are reduced by an acidic environment, further decreasing circulating levels of active vitamin D [18]. Although the DALI trial primarily evaluated lifestyle and vitamin D interventions for gestational diabetes prevention, the role of early pregnancy vitamin D status in maternal lipid metabolism and fetal metabolic exposure remains insufficiently characterized. Prior studies have largely focused on glucose outcomes or late‐pregnancy lipid measurements, with inconsistent findings and limited data on longitudinal lipid changes or neonatal metabolic markers. Moreover, whether baseline vitamin D status—independent of supplementation—relates to metabolic trajectories in high‐risk pregnancies has not been clearly established.

In this secondary analysis of the vitamin D And Lifestyle Intervention for gestational diabetes mellitus prevention (DALI) trial, we aimed to examine whether vitamin D status in early pregnancy (< 20 weeks gestation) is associated with longitudinal changes in maternal lipid metabolism and neonatal metabolic markers in overweight/obese pregnant women. Specifically, we investigated whether 25‐OH‐calciferol deficiency or insufficiency, compared with sufficient concentrations, relates to trajectories of maternal lipids and ketone bodies during pregnancy and to lipid and ketone body concentrations in cord blood.

## 2 Materials and Methods

### 2.1 Study Design and Participants

The vitamin D And LIfestyle for gestational diabetes prevention trial (DALI) was a prospective Europe‐wide multicentre randomized controlled trial, which aimed to investigate interventions starting in early pregnancy to prevent GDM. The effects of lifestyle intervention (healthy eating, physical activity, combined) compared with usual care or vitamin D3 supplementation (with or without lifestyle intervention) compared with placebo (with or without lifestyle intervention) was investigated. in overweight/obese pregnant women at risk for GDM [14, 19, 20].

Inclusion criteria included age 18 years or older, a singleton pregnancy before 20 weeks of gestation, a pre‐pregnancy BMI ≥ 29 kg/m2 and the ability to give informed consent. As DALI was designed to prevent GDM, women diagnosed with GDM at baseline were excluded from the trial based on an oral Glucose Tolerance Test (oGTT) according to the World Health Organization (WHO) 2013 guidelines [21]. Further exclusion criteria were: pre‐existing diabetes, chronic medical conditions defined by the local investigator (e.g., valvular heart disease), psychiatric disorders, inability to safely walk more than 100 m, the need for a complex diet, and/or lack of fluency in the primary language of the recruitment country or inability to converse with the lifestyle coach in any available language of the intervention materials.

All 1045 DALI participants gave their written informed consent and were recruited between 2012 and 2015 in one of the eleven participating centres across nine European countries [United Kingdom, Ireland, Netherlands, Austria, Poland, Italy (Padua and Pisa), Spain, Denmark (Odense and Copenhagen) and Belgium]. The local ethics committees approved the DALI trial. DALI received funding from the European Union 7th framework program (Grant Agreement no. 242187) and was registered in the ISRCTN registry (registration number ISRCTN70595832). The initial DALI protocol included assessment of serum lipid concentrations. The study protocol as well as main trial outcomes were published and can be read in detail elsewhere [14, 19, 20].

### 2.2 Study Procedures and Study Intervention

**FIGURE 1:**
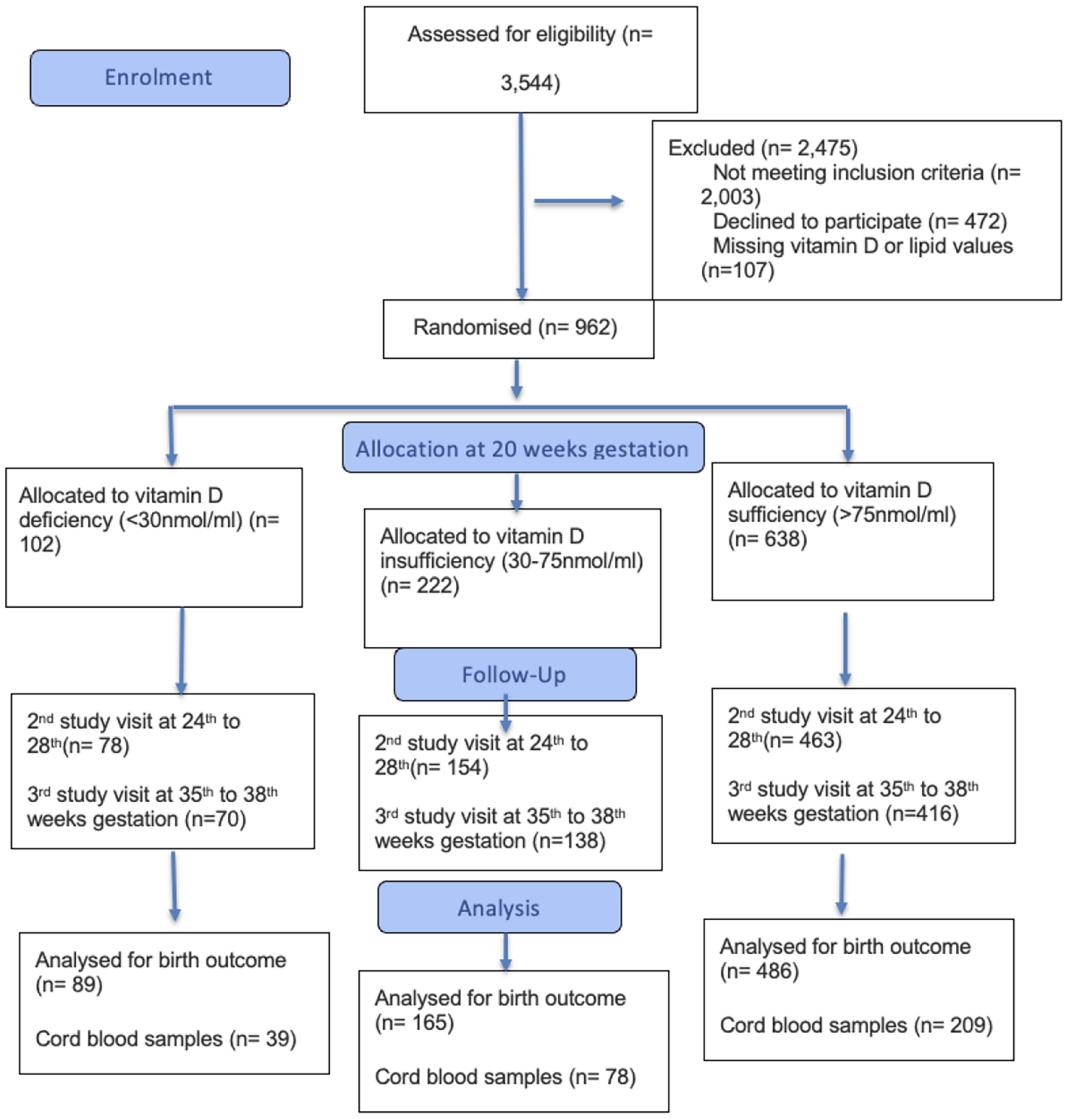
Study flow chart—Report of the recruitment and enrollment of the study participants. wks = weeks.

In this secondary analysis all pregnant women participating in the DALI pilot, lifestyle and vitamin D trials with information on vitamin D concentrations and lipid parameters were included. Study procedures, randomization, blinding and sample size calculations of the DALI trial were described previously [14, 15, 19, 20]. Briefly, obese pregnant women were randomized after a baseline examination to receive either lifestyle advice (healthy eating and/or physical activity) in the lifestyle intervention trials or vitamin D supplementation or placebo combined with and without lifestyle advice in the vitamin D intervention trial. Study visits were performed before 20, at 24–28 and 35–37 weeks of gestation and at delivery. Individuals with missing vitamin D or lipid concentrations were excluded (Figure 1). In total 962 DALI participants were further analysed for this examination.

Based on the baseline total 25‐OH‐calciferol concentrations the pregnant women were divided in vitamin D sufficiency groups with cut offs as follows: total 25‐OH‐calciferol < 30 nmol/L = deficient (*n* = 102), 30–50 nmol/L = insufficient (*n* = 222), > 50 nmol/L = sufficient (*n* = 638). 25‐OH‐calciferol was used as a proxy of vitamin D status. Due to exclusion criteria further women were excluded after baseline oGTT due to GDM diagnosis before 20 weeks gestation, oGTT performed after 20 weeks gestation or not performed at all, BMI < 29 kg/m^2^ or withdrawal of consent, leaving 695 pregnant women for further analysis after week 20 of gestation.

### 2.3 Assessments

Blood samples, anthropometric and functional measurements such as height and weight, were performed at baseline (< 20 weeks), 24–28 and 35–37 weeks of gestation and explained in detail elsewhere [19]. Neonatal skinfolds were measured within 48 h after birth. At the baseline study visit, questionnaires were distributed to the participants to obtain individual information about demographics, pre‐pregnancy weight, smoking habit, past/current medical obstetric, medication history and current medication/multivitamin intake. Venous cord blood was drawn immediately after birth. World Health Organization 2013 GDM criteria (75 g oGTT, diagnostic criteria: fasting > = 5.1 mmol/L (92 mg/dL), 1 h postprandial > = 10.0 mmol (180 mg/dL) and 2 h postprandial glucose > = 8.5 mmol/L (153 mg/dL)) were used to diagnose gestational diabetes mellitus [21]. Lifestyle behaviour was assessed by self‐reporting tools including the Pregnancy Physical Activity Questionnaire, giving approximations of activity and sedentary behaviour, and a 12‐item food intake questionnaire implemented to gather food frequencies and amounts. Nutritional components were calculated as total numbers/week as described earlier and in Table 4 [15].

### 2.4 Laboratory Analyses

All blood samples were drawn in a fasted state, immediately centrifuged, aliquoted and stored at −20°C or −80°C in a central biobank in Graz, Austria. Liquid chromatography mass spectrometry (ClinMass)/mass spectrometry (LCeMS/MS) using a complete kit (RECIPE Chemicals + Instruments GmbH, Munich, Germany) was used to assess serum 25‐OH‐D concentrations, as described in previous publications [14, 15]. The internal standard D6‐25‐OH‐D3 was used and included in the precipitation reagent. Our vitamin D samples were compared with a Vitamin D External Quality Assessment Scheme (DEQAS) certified center for external analysis validation. Due to equipment malfunction in the central laboratory, cord blood, 25‐OH‐D concentrations were measured at a reference laboratory in Barcelona, Spain, also using LCeMs/Ms.

Triglycerides (TG, sensitivity 0.5 mg/dL, intra‐ and inter‐assay coefficients of variability (low/high concentrations) 0.94/0.82%, 1.71/0.99%), HDL cholesterol (HDL‐C, 2 mg/dL 2.1/1.4%, 3.0/2.4%), LDL cholesterol (LDL‐C, calculated by Friedewald formula (LDL‐C = TC − HDL‐C − TG/5)), non‐esterified free fatty acids (FFA, 1.5%), 3‐hydroxy butyrate (3BHB, 0.05 mmol/L, 0.56/0.32%, 2.15/1.93%), leptin (1.0 ng/mL; 6.0/6.9%, 11.6/8.7%), glucose and insulin (0.5 μU/L, 3.3/4.6%, 2.6/5.9%) were analysed as reported in our prior publications [7, 17, 18].

### 2.5 Statistics

Continuous variables were presented as means ± standard deviations and medians for normally distributed data and as median (interquartile range [IQR]) for non‐normally distributed data. Categorical variables are summarized as counts and percentages. Normality was assessed visually using histograms and by evaluating skewness and kurtosis. Variables with skewed distributions were log‐transformed prior to analysis. Transformed variables were used in regression analyses, while descriptive statistics are presented in their original scale. Baseline comparisons across vitamin D status groups (deficient, insufficient, sufficient) were performed using one‐way analysis of variance (ANOVA) with Tukey's post hoc correction for multiple comparisons. Categorical variables were compared using the Chi‐square test.

Changes in maternal lipid and metabolic parameters (triglycerides, LDL‐C, HDL‐C, free fatty acids [FFA], 3‐β‐hydroxybutyrate [3BHB] and leptin) from baseline to 24–28 and 35–37 weeks of gestation were examined using general linear regression models. Each model included the respective baseline lipid value as a covariate to account for initial differences.

Adjustment was performed for the baseline values of the outcome variable, maternal age, body mass index (BMI) at baseline, insulin resistance at time of examination (HOMA Index), self‐reported food intake at time of examination (portion size), self‐reported physical activity, maternal smoking, gestational week, weight gain, ethnicity, educational level and study arm/randomization (fully adjusted model).

A sensitivity analysis was conducted to evaluate the potential influence of exogenous vitamin D_3_ supplementation on the observed associations. Participants assigned to a vitamin D_3_ supplementation arm of the DALI trial (*n* = 79) were excluded. The analyses were repeated in the restricted dataset using the same multivariable‐adjusted regression models as in the primary analysis, including identical covariate adjustments. Adjusted mean differences and 95% confidence intervals were derived to assess the stability of the results.

Dietary components and physical activity were analysed after adjustment for baseline levels using general linear models.

Cord blood lipids were analysed using general linear models with adjustment for gestational age, maternal age, maternal pregestational BMI, maternal baseline levels of the outcome variable, insulin resistance, weight gain, fetal sex, ethnicity, educational level and study arm/randomization. Binary outcomes were analysed using logistic regression, and results are presented as odds ratios (ORs) with 95% confidence intervals (CIs).

No formal correction for multiple testing was applied; therefore, findings should be interpreted cautiously as exploratory.

All statistical analyses were performed using SPSS version 28.0.1.0 (IBM Corp., Armonk, NY, USA) and GraphPad Prism version 10.2.3 (GraphPad Software, San Diego, CA, USA). All tests were two‐sided, and *p* < 0.05 were considered statistically significant.

## 3 Results

In total, 962 overweight/obese pregnant women had full data about vitamin D and lipid concentrations at baseline and were included in this secondary analysis. They were divided into groups with vitamin D deficiency (*n* = 102), insufficiency (*n* = 222), and sufficient vitamin D levels (*n* = 638) (Figure 1). After further exclusion of another 267 participating women, mainly due to positive oGTT results for GDM at baseline, 695 pregnant women were further analysed after 20 weeks gestation.

### 3.1 Baseline Results

Women with sufficient 25‐OH‐calciferol concentrations were older, were more often highly educated and of European descent than women with insufficient or deficient vitamin D status (Table 1). Family members with diabetes were less often observed in the vitamin D sufficient group. Furthermore, women with sufficient 25‐OH‐calciferol concentrations had higher pregnancy vitamin supplement intake, 25‐OH‐colecalciferol (25‐OH‐D3) and 25‐OH‐calciferol (25‐OH‐D) concentrations at baseline.

**TABLE 1:** Baseline characteristics of patients of three different vitamin D sufficiency groups at < 20 weeks: Deficient (25‐OH‐D Total < 30 nmol/L), insufficient (25‐OH‐D Total 30–50 nmol/L), sufficient (25‐OH‐D Total > 50 nmol/L).

	Deficiency *N* = 102			Insufficiency *N* = 222			Sufficiency *N* = 638			*p* for whole analysis
	Median	IQR		Median	IQR		Median	IQR		
Age (y)	30.8*	26.5	36.3	31.7*	28.1	35.0	32.8*	29.0	36.3	0.005
Height (cm)	163***^;§^	158	169	165^§^	160	170	166***	162	170	< 0.001
Gestational week	14.4	12.7	16.7	15.1	13.1	16.9	15.1	13.7	16.7	ns
Weight gain until 1st visit (kg)	2.0	−0.6	4.1	1.5	−0.5	4.4	1.8	−0.4	4.2	ns
Pre pregnancy BMI (kg/m^2^)	34.6***^,§^	31.5	37.7	33.2^§^	30.8	36.3	32.6***	30.2	35.6	< 0.001

	*N*	%	*N*	%	*N*	%	*p*
BMI 25–30 (kg/m^2^)	9/101	8.9	39/220	17.7	134/634	21.1	0.002
BMI 30–35 (kg/m^2^)	46/101	45.5	106/220	48.2	316/634	49.8	
BMI 35–30 (kg/m^2^)	26/101	25.5	55/220	25.0	131/634	20.7	
BMI > 40 (kg/m^2^)	20/101	19.9	20/220	9.1	53/634	8.4	
European descent	63/99	63.6	177/222	79.7	584/636	91.8	< 0.001
Higher education	34/99	34.3	108/222	48.6	380/634	59.9	< 0.001
Multiparity	72/99	72.7	128/222	57.7	397/636	62.4	0.036
Diabetes in family	38/102	37.3	60/222	27.0	139/639	21.8	0.002
Personal history of GDM	11/72	15.3	10/127	7.9	37/389	9.5	ns
Smoking	23/99	23.2	39/222	17.6	99/632	15.7	ns
Taking pregnancy vitamins	64/102	62.7	174/222	78.4	581/639	90.9	< 0.001

	Median	IQR		Median	IQR		Median	IQR		*p*
TG (mmol/L)	1.26	1.00	1.60	1.24	1.00	1.61	1.31	1.02	1.66	ns
LDL‐C (mmol/L)	2.90	2.40	3.40	2.90*	2.40	3.40	3.00*	2.60	3.60	0.016
HDL‐C (mmol/L)	1.34	1.19	1.54	1.40	1.22	1.60	1.40	1.24	1.58	ns
TG/HDL ratio	2.06	1.60	2.84	1.98	1.52	2.88	2.12	1.61	2.84	ns
FFA (mmol/L)	0.69^§^	0.56	0.82	0.62^§^	0.49	0.76	0.61	0.49	0.77	0.033
3BHB (mmol/L)	0.100**^,§§^	0.050	0.100	0.100^§§^	0.000	0.100	0.100**	0.000	0.100	0.005
Leptin (ng/dL)	34.1	22.8	43.3	35.9	26.2	45.4	32.1	23.4	44.4	ns
HOMA IR	3.16*	2.09	4.75	2.99	2.11	4.25	2.70*	1.99	3.74	0.007
25‐OH‐D3 (nmol/L)	22.1***^,§§§^	17.5	25.8	40.4***^,§§§^	34.6	44.7	69.4***^,§§§^	60.3	85.9	< 0.001
25‐OH‐D Total (nmol/L)	23.3***^,§§§^	18.3	26.0	41.7***^,§§§^	35.3	45.8	71.8***^,§§§^	61.9	88.7	< 0.001

*Note:* Subgroup analysis: Sufficiency vs. other groups: * < 0.05, ** < 0.01, *** < 0.001, Deficiency vs. other groups: § < 0.05, §§ < 0.01, §§§ < 0.001.

Abbreviations: 3BHB, 3 hydroxybutyrate; BMI, body mass index; BP, blood pressure; dia, diastolic; FFA, free fatty acids; GDM, gestational diabetes; HOMA IR, Homeostatic Model Assessment for Insulin Resistance; ns, not significant; PCOS, polycystic ovary syndrom; sys, systolic; TG, triglycerides.

At baseline, women with sufficient levels had significantly lower BMI, 3BHB and insulin resistance compared with women with deficient 25‐OH‐calciferol concentrations (Table 1). Distribution of vitamin D sufficiency across BMI groups is shown in Table 1.

Women staying vitamin D deficient throughout pregnancy (*n* = 29) from baseline until 35–37 weeks of gestation were not showing any significant differences in any baseline characteristics (anthropometric, sociodemographic or metabolic) compared with pregnant women changing to vitamin D insufficiency (*n* = 25) or sufficiency (*n* = 8) (numbers not shown in text or table). Significant differences between these groups were seen in changes of 25‐OH‐calciferol concentrations (change in 25‐OH‐D from baseline until 35–37 weeks of gestation: deficiency staying deficient: mean ± SD: −1.67 ± 6.60 nmol/L; deficiency changing to insufficiency 29.16 ± 12.44 nmol/L, deficiency changing to sufficiency: 70.52 ± 20.66 nmol/L, *p* < 0.01).

**FIGURE 2:**
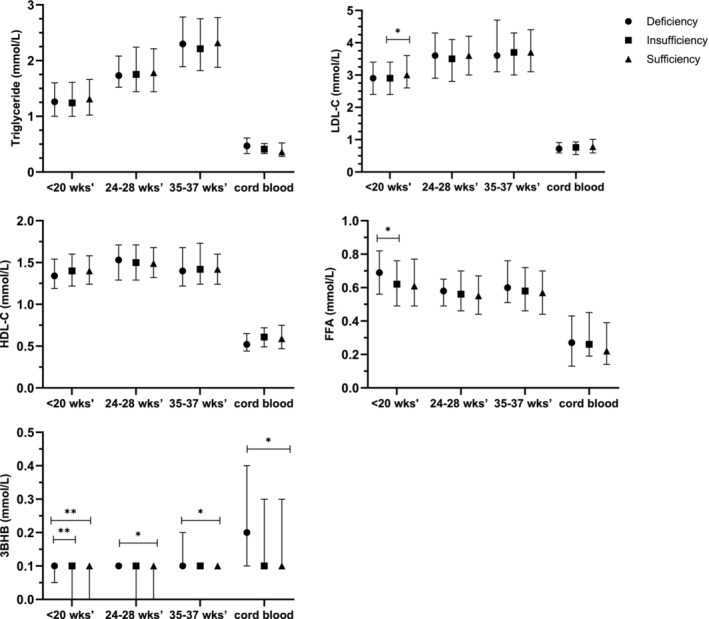
Triglyceride, LDL‐C, HDL‐C, FFA and ß OH butyrate concentrations throughout pregnancy and in cord blood. In the fully adjusted model significantly larger adjusted mean differences in LDL‐C and 3BHB concentrations and smaller adjusted mean differences in 25‐OH‐D3 were observed in the deficient vitamin D group compared with the sufficient group (Table 2). No differences were found between vitamin D sufficient and insufficient groups. Also, additional adjustment for pregnancy vitamin intake did not change results. Levels of significance * < 0.05, ** < 0.01.

Women with sufficient 25‐OH‐calciferol concentrations had higher LDL‐C than women with insufficient vitamin D at baseline, which was not observed comparing sufficient with deficient vitamin D groups (Table 1). However, HDL‐C, FFA and triglyceride concentrations did not differ (Figure 2).

### 3.2 24–28. Weeks' Gestation

Absolute concentrations of lipid parameters (especially triglycerides and LDL‐C) and 3BHB increased throughout pregnancy (Figure 2). At 24–28 weeks' gestation, mean lipid concentrations were similar between groups (Figure 2, Table 2), but significantly higher 3BHB concentrations were found in the deficient versus sufficient vitamin D groups.

**TABLE 2:** Adjusted mean differences of triglycerides, LDL‐C, HDL‐C, FFA, and ß OH butyrate and leptin at 24–28 and 35–37 weeks of gestation between vitamin D sufficiency groups defined as per vitamin D status before 20 weeks.

	Deficiency *N* = 78			Insufficiency *N* = 154			Sufficiency *N* = 463			*p*	Adj. mean difference (95% CI, *p*‐value) Sufficiency versus deficiency	*p*	Adj. mean difference (95% CI, *p*‐value) Sufficiency versus insufficiency	*p*
	Median	IQR		Median	IQR		Median	IQR						
24–28 wks' gestation														
TG (mmol/L)	1.73	1.52	2.08	1.75	1.44	2.24	1.78	1.44	2.21	ns	0.09 (−0.06; 0.24)	ns	0.01 (−0.09; 0.12)	ns
LDL‐C (mmol/L)	3.60	2.90	4.30	3.50	2.80	4.10	3.60	3.00	4.20	ns	−0.29 (−0.51; −0.07)	0.011	−0.11 (−0.27, 0.05)	ns
HDL‐C (mmol/L)	1.53	1.29	1.71	1.50	1.29	1.71	1.49	1.32	1.68	ns	−0.04 (−0.09; 0.02)	ns	0.02 (−0.03; 0.06)	ns
TG/HDL ratio	1.18	0.94	1.41	1.17	0.83	1.59	1.19	0.90	1.57	ns	0.21 (−0.08;0.50)	ns	−0.02 (−0.23;0.19)	ns
FFA (mmol/L)	0.58	0.49	0.65	0.56	0.46	0.70	0.55	0.44	0.67	ns	0.01 (−0.04; 0.07)	ns	−0.01 (−0.06; 0.03)	ns
3BHB (mmol/L)	0.100*	0.100	0.100	0.100	0.000	0.100	0.100*	0.000	0.100	0.009	−0.026 (−0.048;‐0.005)	0.015	−0.014 (−0.030; 0.001)	ns
Leptin (ng/dl)	31.8	24.0	46.3	34.3	26.4	45.3	32.7	24.0	42.7	ns	3.1 (−0.4; 6.6)	ns	2.3 (−0.2; 4.9)	ns
HOMA IR	3.35	2.24	4.38	3.18	2.40	4.21	2.92	2.20	4.00	ns	0.02 (−0.41;0.46)	ns	−0.16 (−0.49;0.16)	ns
25‐OH‐D3 (nmol/L)	30.1***^,§§§^	19.8	47.2	51.0***^,§§§^	37.7	72.5	80.4***^,§§§^	57.0	105.0	< 0.001	16.4 (4.2; 28.5)	0.009	7.6 (−0.9; 16.0)	ns
25‐OH‐D Total (nmol/L)	31.6***^,§§§^	20.4	47.2	51.9***^,§§§^	38.2	74.4	84.1***^,§§§^	59.5	106.0	< 0.001	8.0 (−3.3; 11.5)	ns	4.1 (−3.3; 11.5)	ns
Weight gain (kg)	3.8	1.4	6.0	3.4	1.7	5.8	3.7	2.0	5.6	ns	−0.5 (−1.3; 0.2)	ns	0.0 (−0.6; 0.6)	ns

	Deficiency *N* = 70			Insufficiency *N* = 138			Sufficiency *N* = 416			*p*	Adj. mean difference (95% CI, *p*‐value) Sufficiency versus deficiency	*p*	Adj. mean difference (95% CI, *p*‐value) Sufficiency versus insufficiency	*p*
	Median	IQR		Median	IQR		Median	IQR						
35–37 wks' gestation														
TG (mmol/L)	2.30	1.89	2.78	2.21	1.82	2.75	2.32	1.88	2.77	ns	0.00 (−0.21; 0.22)	ns	−0.05 (−0.21; 0.10)	ns
LDL‐C (mmol/L)	3.60	3.10	4.70	3.70	3.00	4.30	3.70	3.10	4.40	ns	−0.32 (−0.60; −0.04)	0.025	−0.21 (−0.41; −0.01)	0.039
HDL‐C (mmol/L)	1.40	1.22	1.68	1.42	1.24	1.73	1.42	1.24	1.60	ns	0.05 (−0.04; 0.13)	ns	0.01 (−0.05; 0.07)	ns
TG/HDL ratio	1.18	0.94	1.41	1.17	0.83	1.59	1.19	0.90	1.57	ns	−0.1 (−0.6; 0.3)	ns	−0.1 (−0.4; 0.3)	ns
FFA (mmol/L)	0.60	0.51	0.76	0.58	0.46	0.72	0.57	0.44	0.70	ns	−0.03 (−0.10; 0.04)	ns	−0.03 (−0.07; 0.02)	ns
3BHB (mmol/L)	0.100*	0.100	0.200	0.100	0.100	0.100	0.100*	0.100	0.100	0.013	−0.026 (−0.062; 0.011)	ns	−0.018 (−0.044; 0.008)	ns
Leptin (ng/dl)	34.8	27.6	47.9	31.8	23.6	46.3	32.6	23.4	45.,6	ns	−0.9 (−5.2; 3.5)	ns	1.7 (−1.6; 4.9)	ns
HOMA IR	3.78*	2.57	5.09	3.56	2.88	4.95	3.19*	2.43	4.33	0.011	−0.21 (−1.35; 0.93)	ns	−0.80 (−1.63, 0.04)	ns
25‐OH‐D3 (nmol/L)	37.4***^,§§§^	19.8	58.7	52.8***^,§§§^	36.4	79.4	77.4***^,§§§^	49.6	104.2	< 0.001	16.4 (2.0; 30.8)	0.025	2.8 (−7.1; 12.8)	ns
25‐OH‐D Total (nmol/L)	39.6***^,§§§^	20.3	58.8	54.8***^,§§§^	37.0	85.3	79.2***^,§§§^	53.1	107.4	< 0.001	13.6 (−1.0; 28.2)	ns	0.5 (−9.6;10.6)	ns
Weight gain (kg)	7.0	4.5	11.3	8.00	5.7	11.1	7.5	4.7	10.7	ns	−0.8 (−2.2; 0.7)	ns	−0.5 (−1.5; 0.6)	ns

*Note:* Sufficiency versus other groups: levels of significance * < 0.05, *** < 0.001, Deficiency versus other groups: levels of significance §§§ < 0.001.

Abbreviations: 3BHB, 3 hydroxybutyrate; FFA, free fatty acids; HOMA IR, Homeostatic Model Assessment for Insulin Resistance; ns, not significant; TG, triglycerides; wks, weeks.

In the fully adjusted regression model, the vitamin D–deficient group exhibited significantly larger adjusted mean differences in LDL‐C and 3BHB concentrations, and smaller adjusted mean differences in 25(OH)D_3_ levels, compared with the vitamin D–sufficient group (Table 2). No significant differences were observed between the vitamin D–sufficient and –insufficient groups. Further adjustment for prenatal vitamin intake did not materially alter these results. No differences were found in other lipid or metabolic parameters such as HDL‐C, triglycerides, leptin or HOMA‐IR.

A sensitivity analysis, excluding women receiving vitamin D_3_ supplementation (*n* = 79) in one of the DALI trial intervention arms, yielded consistent results. Significantly lower adjusted mean differences in LDL‐C (−0.28; 95% CI: −0.50, −0.06) and 3BHB (−0.032; 95% CI: −0.062, −0.002) concentrations were observed when comparing vitamin D–sufficient with –deficient groups. Additionally, a significant difference in 3BHB concentrations was observed when comparing vitamin D–sufficient with –insufficient groups (−0.016; 95% CI: −0.032, −0.001), whereas no significant difference was found for LDL‐C (−0.06; 95% CI: −0.23, 0.11).

### 3.3 35–37. Weeks' Gestation

At 35–37 weeks of gestation, lipid concentrations were comparable across groups; however, the vitamin D–deficient group exhibited significantly higher mean 3BHB concentrations and higher HOMA‐IR compared with the vitamin D–sufficient group (Figure 2, Table 2).

After full adjustment, adjusted mean differences in LDL‐C concentrations were significantly larger in the vitamin D–deficient group compared with the vitamin D–sufficient group. No significant differences were observed for other lipid parameters or 3BHB. Additional adjustment for prenatal vitamin intake did not materially alter these results.

The sensitivity analysis, excluding women receiving vitamin D_3_ supplementation in one of the DALI trial intervention arms demonstrated significantly lower adjusted mean differences in LDL‐C (−0.36; 95% CI: −0.64, −0.07) and 3BHB (−0.032; 95% CI: −0.062, −0.002) concentrations when comparing vitamin D–sufficient with –deficient groups. Similar differences were also observed when comparing vitamin D–sufficient with –insufficient groups for LDL‐C (−0.23; 95% CI: −0.44, −0.02) and 3BHB (−0.025; 95% CI: −0.047, −0.003).

**FIGURE 3:**
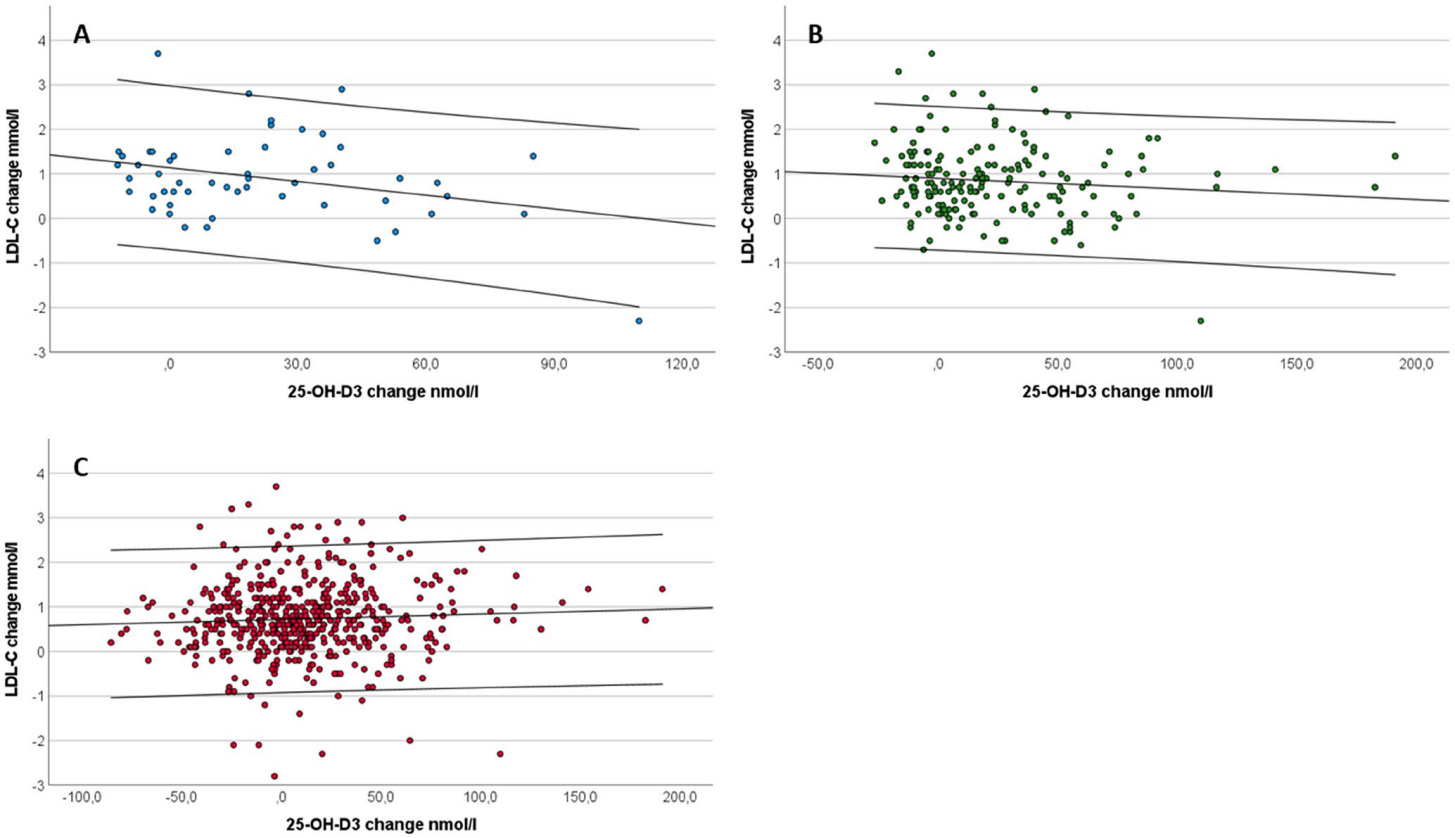
Correlations of changes in 25‐OH‐D3 and LDL‐C throughout pregnancy in the different vitamin D sufficiency groups (A—vitamin D deficiency, blue; B—vitamin D insufficiency, green; C—vitamin D sufficiency, blue).

Figure 3 shows correlations between changes in LDL‐C and 25‐OH‐D3 from baseline to 35–37 weeks of gestation across vitamin D groups. A negative correlation was observed in the vitamin D deficiency group (A, blue coloured dots) with increasing 25‐OH‐D3 concentrations associated with decreasing LDL‐C concentrations, which was not found t in insufficiency (B—green coloured dots) or sufficiency groups (C‐ red coloured dots).

### 3.4 Cord Blood

In cord blood, mean 25‐OH‐D3 and total 25‐OH‐D concentrations were significantly higher in infants of vitamin D–sufficient mothers compared with those of vitamin D–deficient mothers, with maternal status defined before 20 weeks' gestation. 3BHB concentrations were significantly higher in the deficient vitamin D group versus the sufficient group (Table 3). Cord blood triglyceride, LDL‐C, HDL‐C and FFA concentrations and birth and pregnancy outcomes were similar between groups.

**TABLE 3:** Adjusted mean differences between vitamin D sufficiency groups at < 20 weeks in fetal cord blood, birth and pregnancy outcomes.

Birth outcome	Deficiency *N* = 39			Insufficiency *N* = 78			Sufficiency *N* = 209			*p*	Adj. mean difference (95% CI, *p*‐value) Sufficiency versus deficiency	*p*	Adj. mean difference (95% CI, *p*‐value) Sufficiency versus insufficiency	*p*
	Median	IQR		Median	IQR		Median	IQR						
Gestational age at birth (wks)	40.3	38.9	41.4	39.7	38.9	40.9	39.7	38.7	40.7	ns	−0.4 (−1.6; 0.8)	ns	0.3 (−0.5; 1.2)	ns
TG (mmol/L)	0.47	0.33	0.61	0.41	0.33	0.51	0.36	0.28	0.52	ns	−0.04 (−0.25; 0.17)	ns	−0.05 (−0.22; 0.11)	ns
LDL‐C (mmol/L)	0.72	0.59	0.91	0.76	0.54	0.93	0.78	0.59	1.01	ns	0.04 (−0.29; 0.37)	ns	0.07 (−0.19; 0.33)	ns
HDL‐C (mmol/L)	0.52	0.44	0.65	0.61	0.49	0.72	0.59	0.47	0.75	ns	0.04 (−0.10; 0.17)	ns	0.03 (−0.08; 0.14)	ns
TG/HDL ratio	0.87	0.57	1.36	0.67	0.50	0.99	0.61	0.44	0.89	ns	−0.13 (−0.95; 0.68)	ns	−0.23 (−0.87; 0.41)	ns
FFA (mmol/L)	0.27	0.13	0.43	0.26	0.19	0.45	0.22	0.14	0.39	ns	−0.01 (−0–12; 0.10)	ns	−0.09 (−0.17; 0.01)	ns
3BHB (mmol/L)	0.200**	0.100	0.400	0.100	0.100	0.300	0.100**	0.100	0.300	0.003	−0.156 (−0.258; −0.053)	0.003	−0.096 (−0.176; −0.015)	0.021
Leptin (ng/dL)	7.5	4.4	13.3	8.3	4.0	15.4	8.1	4.6	13.8	ns	−0.3 (−4.3; 3.9)	ns	−1.1 (−4.2; 2.0)	ns
C‐pep/Gluc ratio	0.15	0.11	0.22	0.15	0.11	0.23	0.16	0.10	0.23	ns	0.02 (−0.03; 0.07)	ns	0.00 (−0.03; 0.04)	ns
Neonatal skinfolds (mm)	19.8	18.6	23.0	20.8	17.0	24.4	20.3	17.4	23.6	ns	0.3 (−1.7; 2.3)	ns	−0.7 (−2.2; 0.8)	ns
Abd. Circumf (cm)	32.6	30.0	34.5	33.0	31.5	35.0	33.6	31.5	35.0	ns	−0.1 (−1.3; 1.0)	ns	0.3 (−1.2; 0.4)	ns
25‐OH‐D3 (nmol/L)	15.0***^,§§§^	0.0	30.0	37.5***^,§§§^	22.6	59.4	52.5***^,§§§^	35.0	70.0	< 0.001	16.9 (3.3; 30.4)	0.015	1.8 (−7.4; 11.1)	ns
25‐OH‐D total (nmol/L)	15.0***^,§§§^	0.0	30.0	37.5***^,§§§^	22.6	59.4	52.5***^,§§§^	35.0	70.0	< 0.001	14.9 (1.2; 28.6)	0.033	0.3 (−9.1; 9.7)	ns

Perinatal outcome	Deficiency		Insufficiency		Sufficiency			OR sufficiency versus deficiency 95% CI		OR sufficiency versus insufficiency 95% CI	
	*N*	%	*N*	%	*N*	%					
Female sex	22/39	56.4	31/78	39.7	106/209	50.7	ns	1.26	0.63; 2.50	0.64	0.38; 1.09
SGA	3/39	7.7	2/76	2.6	17/205	8.3	ns	0.92	0.26; 3.31	0.30	0.07; 1.33
LGA	6/39	15.4	10/76	13.2	26/205	12.7	ns	1.25	0.48; 3.28	1.04	0.48; 2.28
preeclampsia	0/38	0	5/76	6.6	4/201	2.0	ns	N/A	N/A	3.47	0.91; 13.28
PIH	2/38	5.3	11/76	14.5	31/201	15.4	ns	0.31	0.07; 1.33	0.93	0.44; 1.96
Preterm delivery	2/39	5.1	5/76	5.1	4/209	1.9	ns	2.77	0.49; 15.68	2.85	0.69; 11.68
C‐section	13/39	33.3	25/77	32.5	74/208	35.6	ns	0.91	0.44; 1.87	0.87	0.50; 1.52
Hyperbilirubinemia	1/39	2.6	3/68	4.4	7/185	3.8	ns	0.67	0.08; 5.60	1.17	0.30; 4.67
NICU	2/38	5.3	6/73	8.2	16/194	8.2	ns	0.62	0143; 2.81	0.99	0.37; 2.65

*Note:* Sufficiency versus other groups: levels of significance ** < 0.01, *** < 0.001, Deficiency versus other groups: levels of significance §§§ < 0.001.

Abbreviations: 3BHB, 3OH butyrate; abd. circumf, abdominal circumference; C‐pep/Gluc ratio, c‐peptide/glucose ratio; c‐section, caesarean section; FFA, free fatty acids; LGA, large for gestational age; neonatal skinfolds, sum of neonatal skinfolds; NICU, neonatal intensive care unit; ns, not significant; PIH, Pregnancy induced hypertension; SGA, small for gestational age; TG, triglycerides.

Compared with the vitamin D–sufficient group, the maternal vitamin D–deficient group exhibited significantly higher adjusted mean 3BHB concentrations and significantly lower 25‐OH‐D3 and total 25‐OH‐D concentrations.

### 3.5 Nutrient Intake and Physical Activity

At baseline, women in the vitamin D–sufficient group had higher fibre and protein intake compared with the vitamin D–deficient group (Table 4). At 24–28 weeks of gestation, no significant differences in nutrient intake were observed between groups. However, at 35–37 weeks, protein intake remained significantly higher in the vitamin D–sufficient group.

**TABLE 4:** Nutrient intake and physical activity before 20, at 24–28 and 35–37 weeks of gestation.

< 20 weeks' gestation	Deficiency *N* = 86		Insufficiency *N* = 209		Sufficiency *N* = 587		*p*
	Mean (SD)		Mean (SD)		Mean (SD)		
Sugar drinks (*n*/week)	9.4	14.9	6.9	8.3	7.1	9.9	ns
Fibre (*n*/week)	30.1	19.1	28.4*	20.5*	33.7*	25.7*	0.02
Protein (*n*/week)	7.6*	6.8	8.5	6.1	10.0*	11.1	0.028
Fat (*n*/week)	5.5	5.4	6.4	5.7	7.1	10.5	ns
Carbohydrates (*n*/week)	41.7	26.1	37.3	23.4	42.6	29.1	ns
Portion size (*n*/week)	23.0	17.3	20.7	14.3	21.5	17.3	ns
Total PA, MET.h/wk	169.1	76.2	177.8	102.0	176.0	94.9	ns
Sedentary time, MET.h/wk	14.0	9.7	14.7**	10.0	12.5**	8.6	0.007
MVPA, MET.h/wk	60.6	50.0	62.6	63.8	69.2	68.6	ns

24–28 weeks' gestation	Deficiency *N* = 75		Insufficiency *N* = 140		Sufficiency *N* = 424			Mean difference (95% CI, *p*‐value) sufficiency versus deficiency		Mean difference (95% CI, *p*‐value) sufficiency versus insufficiency	
Sugar drinks (*n*/week)	6.6	8.8	5.3	11.7	5.1	8.1	ns	−1.1 (−3.1; 0.9)	ns	0.4 (−1.1; 1.9)	ns
Fibre (*n*/week)	34.6	21.6	35.9	33.1	36.7	21.5	ns	0.9 (−5.0; 6.9)	ns	−1.0 (−5.5; 3.5)	ns
Protein (*n*/week)	8.6	7.7	8.8	7.1	9.0	5.9	ns	0.6 (−1.0; 2.2)	ns	−0.1 (−1.2; 1.1)	ns
Fat (*n*/week)	7.0	6.2	5.4	4.8	6.0	5.8	ns	−0.7 (−2.1; 0.6)	ns	0.6 (−0.4; 1.6)	ns
Carbohydrates (*n*/week)	39.6	25.9	35.0	26.2	36.9	24.4	ns	−3.8 (−10.2; 2.5)	ns	0.3 (−4.3; 5.0)	ns
Portion size (*n*/week)	20.0	14.3	17.3	15.5	18.1	14.9	ns	−1.2 (−5.0; 2.6)	ns	0.5 (−2.3; 3.3)	ns
Total PA, MET.h/wk	168.6	96.8	168.5	90.5	159.6	79.9	ns	−11.0 (−27.4; 5.4)	ns	−5.0 (−17.4; 7.4)	ns
MVPA, MET.h/wk	58.2	59.1	59.6	58.3	57.6	54.0	ns	−3.1 (−14.2; 8.1)	ns	−3.2 (−11.6; 5.2)	ns
Sedentary time, MET.h/wk	14.1*	9.8	13.3	9.3	11.6*	8.0	0.017	−1.5 (−3.3; 0.3)	ns	−0.7 (−2.0; 0.7)	ns

35–37 weeks' gestation	Deficiency *N* = 65		Insufficiency *N* = 128		Sufficiency *N* = 383						
Sugar drinks (*n*/week)	6.0	10.6	5.0	10.7	4.8	6.8	ns	−0.7 (−2.9; 1.5)	ns	0.8 (−2.0; 1.2)	ns
Fibre (*n*/week)	32.8	20.6	31.4	20.5	36.1	21.2	ns	2.7 (−2.7; 8.1)	ns	2.9 (−1.1; 6.9)	ns
Protein (*n*/week)	6.7**	3.3	8.2	5.7	9.1**	6.3	0.006	2.3 (0.6; 3.9)	0.008	0.7 (−0.5; 1.9)	ns
Fat (*n*/week)	5.2	4.9	6.7	7.7	6.2	6.0	ns	1.1 (−0.6; 2.8)	ns	−0.6 (−1.9; 0.6)	ns
Carbohydrates (*n*/week)	35.5	24.3	33.4	28.8	36.1	19.9	ns	0.2 (−5.8; 6.3)	ns	1.3 (−3.1; 5.6)	ns
Portion size (*n*/week)	18.3	16.4	17.9	15.7	17.8	12.2	ns	0.6 (−2.6; 2.5)	ns	0 (−2.6; 2.5)	ns
Total PA, MET.h/wk	137.0	88.3	137.6	78.8	131.9	68.6	ns	−6.5 (−22.8; 9.7)	ns	−1.5 (−13.9; 10.8)	ns
MVPA, MET.h/wk	45.7	60.7	44.0	48.1	39.6	41.0	ns	−7.6 (−17.8; 2.5)	ns	−4.3 (−12.0; 3.4)	ns
Sedentary time, MET.h/wk	12.0	8.2	13.2	8.5	12.9	8.8	ns	1.8 (−0.2; 3.8)	ns	0.8 (−0.7; 2.3)	ns

*Note:* Level of significance: levels of significance * < 0.05, ** < 0.01, PA = physical activity, Definition of calculation of food scores for each time point (*n*/week) = frequency per day × times per week; sugar drinks: sugar drinks total = fruit juice total + soft drink total; fibre: total fibre = vegetables total + fruit total + whole‐grain bread total; protein: protein total = meat and eggs total + fish total; fat: fat total = high‐fat milk total + cakes and muffins total + fast food total; carbohydrates: carbohydrates total = cakes and muffins total + fruit total + whole‐grain bread total + fruit juice total + soft drink total + potatoes/pasta total; portion size: portion size total = cakes and muffins total + high‐fat milk total + fruit juice total + soft drink total + potatoes/pasta total.

Abbreviation: MVPA, moderate to vigorous physical activity.

In terms of physical activity, women in the vitamin D–sufficient group exhibited less sedentary behaviour at baseline, a pattern that persisted at 24–28 weeks of gestation.

After adjustment for baseline parameters, the adjusted mean protein intake remained significantly higher in the vitamin D–sufficient group compared with the deficient group. In contrast, the adjusted mean difference in sedentary behaviour was no longer statistically significant between groups.

**FIGURE 4:**
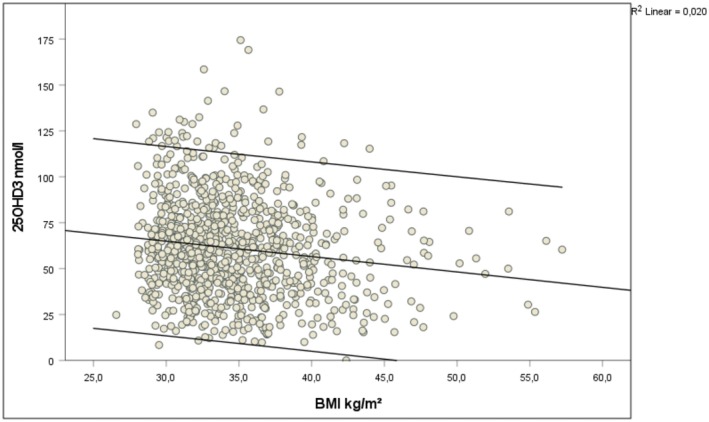
Correlation of baseline maternal 25OHD3 and maternal BMI.

Negative correlations were found between maternal baseline BMI and 25‐OH‐D3 concentrations before 20 weeks gestation (*r* = −0.13, *p* < 0.001), 24–28 weeks gestation (*r* = −0.14, *p* < 0.001) and 35–37 weeks gestation (*r* = −0.16, *p* < 0.001) as well as cord blood (*r* = −0.16, *p* < 0.01) (Figure 4, Table 5).

**TABLE 5:** Correlation matrix of maternal BMI at baseline and 25OHD3 at several timepoints in pregnancy and in cord blood.

Maternal BMI	25OHD3 < 20 weeks	25OHD3 24–28 weeks	25OHD3 35–37 weeks	Cord blood
Correlation	−0.128	−0.140	−0.163	−0.155
*p*	< 0.001	< 0.001	< 0.001	0.005

Before 20 weeks of gestation and at 35–37 weeks of gestation, vitamin D deficiency was associated with HOMA IR (*r* = 0.11, *p* < 0.001 and *r* = 0.13, *p* < 0.01), insulin secretion parameters (Stumvoll 1st phase index: *r* = 0.12; *p* < 0.001 and *r* = 0.14, *p* < 0.01), and 3BHB concentrations (*r* = 0.07, *p* < 0.05 and *r* = 0.15; *p* < 0.001).

## 4 Discussion

This secondary analysis suggests that early pregnancy vitamin D deficiency in women with overweight or obesity is associated with greater increases in LDL cholesterol and ketone body concentrations across gestation, as well as higher cord blood ketone levels in offspring. Importantly, absolute lipid concentrations at individual time points were largely similar between vitamin D groups, indicating that vitamin D status may influence metabolic trajectories rather than static lipid levels. These findings support a potential role of early micronutrient status in shaping metabolic adaptations during pregnancy; however, causality cannot be inferred.

Pregnancy is characterized by progressive hyperlipidemia driven by hormonal changes, increasing insulin resistance and alterations in lipoprotein metabolism [22, 23, 24]. In our cohort, lipid levels increased across all vitamin D groups, consistent with these physiological adaptations. However, women with vitamin D deficiency exhibited a greater increase in LDL cholesterol, suggesting that vitamin D status may modulate the magnitude of lipid changes rather than baseline concentrations.

These findings are in line with previous studies reporting associations between vitamin D status and lipid parameters during pregnancy. For example, vitamin D concentrations were correlated with triglycerides and cholesterol in a cohort of Saudi Arabian women in early pregnancy [24]. However, the general increase in lipids during pregnancy complicates interpretation of cross‐sectional associations [24]. Differences in study populations, including ethnicity and obesity status, may further contribute to inconsistent findings.

Our results are consistent with those of Lepsch et al. [12], who also reported greater increases in LDL‐C (as well as triglycerides and triglyceride/HDL‐C ratio) throughout gestation in women with inadequate vitamin D status, despite no significant differences in absolute lipid concentrations at individual time points. Together, these findings highlight the importance of early pregnancy vitamin D status for subsequent lipid trajectories.

Recent metabolomic studies provide additional insight into potential mechanisms, showing that vitamin D deficiency is associated with alterations in lipid‐related metabolites and pathways involved in energy metabolism, including glycerophospholipid metabolism and CoA biosynthesis [25]. This supports the concept that vitamin D may modulate lipid metabolism at a pathway level, which could explain the differences in lipid trajectories observed in our study despite similar absolute lipid concentrations.

The mechanisms linking vitamin D deficiency to altered lipid metabolism remain incompletely understood, but several biologically plausible pathways exist. Vitamin D signalling via the vitamin D receptor (VDR) may influence hepatic lipid metabolism, including cholesterol synthesis and clearance. In addition, vitamin D deficiency has been associated with impaired insulin sensitivity, as well as chronic low‐grade inflammation and oxidative stress, all of which can adversely affect lipid handling and promote a more atherogenic lipid profile [26, 27, 28].

Pregnancy itself is characterized by progressive insulin resistance and increased lipid mobilization to support fetal growth, resulting in elevated circulating free fatty acids and lipoproteins [29]. In this metabolically dynamic state, vitamin D deficiency may exacerbate these physiological adaptations, thereby amplifying the rise in LDL cholesterol observed in our cohort.

Recent evidence further suggests that vitamin D deficiency during early developmental periods, including pregnancy, may influence metabolic programming and contribute to long‐term alterations in glucose and lipid metabolism through mechanisms involving insulin resistance and VDR‐mediated regulation of metabolic pathways [30]. These findings align with the developmental origins of health and disease (DOHaD) framework, which proposes that early‐life environmental exposures, including maternal micronutrient status, can shape long‐term metabolic health trajectories in the offspring [31].

Intervention studies provide further indirect support for a role of vitamin D in metabolic regulation. In pregnant women with gestational diabetes, combined vitamin D and omega‐3 fatty acid supplementation has been associated with improvements in glucose and lipid parameters, potentially mediated by enhanced insulin receptor expression and calcium‐dependent metabolic pathways [32]. However, these findings cannot be directly translated to our cohort, and the specific contribution of vitamin D remains difficult to isolate.

We observed higher β‐hydroxybutyrate concentrations in vitamin D deficient women at 24–28 weeks of gestation, representing one of the most notable findings of this study. Pregnancy is characterized by a progressive increase in insulin resistance, particularly in the second and third trimesters, promoting lipolysis and hepatic ketogenesis [29]. Vitamin D deficiency has been associated with impaired insulin secretion and increased insulin resistance, which may further enhance this metabolic shift. Reduced vitamin D availability may therefore exacerbate lipolysis and ketone production, resulting in higher circulating β‐hydroxybutyrate concentrations.

Consistent with this hypothesis, we observed associations between vitamin D deficiency and markers of insulin resistance and ketone body concentrations at different time points during pregnancy. Although these associations were relatively weak and not consistently present across all gestational stages, they support the concept that vitamin D status may modulate metabolic flexibility during pregnancy. To our knowledge, the relationship between vitamin D status and ketone body concentrations in pregnancy has not been well described, highlighting a potentially novel metabolic pathway that warrants further investigation.

Maternal vitamin D status not only affects maternal metabolism but may also influence fetal metabolic exposure. In our study, higher cord blood β‐hydroxybutyrate concentrations were observed in offspring of vitamin D deficient women, whereas lipid concentrations were similar across groups. This discrepancy may reflect differences in placental transport mechanisms. Lipid transfer is tightly regulated and dependent on placental processing, whereas ketone bodies can more readily cross the placenta and serve as an alternative energy substrate for the fetus.

Previous studies have highlighted the potential relevance of ketone exposure during fetal development. Elevated maternal ketone levels have been associated with adverse neurodevelopmental outcomes, including lower offspring IQ and impaired motor function [33, 34]. While ketone bodies are a physiological energy source during pregnancy, excessive exposure may reflect altered metabolic conditions. Our findings therefore raise the possibility that vitamin D deficiency could influence the fetal metabolic environment through increased ketone availability.

Vitamin D deficiency was associated with higher BMI, adiposity and markers of insulin resistance in our cohort. Given the well‐established relationships between obesity, vitamin D deficiency and dyslipidemia, residual confounding cannot be fully excluded despite adjustment. Circulating 25‐OH‐calciferol concentrations are inversely associated with multiple measures of adiposity, including BMI, total fat mass, subcutaneous and visceral fat, and waist circumference, consistent with our findings [35]. Several mechanisms may contribute to lower vitamin D levels in obesity. These include reduced mobilization of vitamin D from adipose tissue due to impaired lipolysis, as well as decreased hepatic synthesis of 25‐OH‐calciferol, potentially mediated by negative feedback from elevated circulating 1,25‐dihydroxyvitamin D [36, 37]. Obesity is also strongly associated with dyslipidemia, partly driven by insulin resistance [38]. Insulin plays a central role in lipid metabolism by inhibiting lipolysis in adipose tissue through suppression of hormone‐sensitive lipase, thereby regulating the release of free fatty acids and subsequent lipid handling [38]. This highlights the difficulty of disentangling the independent effects of vitamin D status from underlying metabolic risk.

In addition, socioeconomic factors such as educational level and migration status were associated with vitamin D deficiency, consistent with previous reports [39, 40, 41]. These factors may further influence metabolic health and should be considered when interpreting our findings.

## 5 Limitations

This analysis included participants from intervention groups receiving lifestyle intervention and/or vitamin D supplementation. However, no significant differences in metabolic parameters were observed between the intervention groups and adjustments were made accordingly.

The study population consisted of overweight/obese pregnant women with normal glucose tolerance at baseline, limiting generalizability to other populations. Baseline differences in characteristics such as ethnicity, education, age and LDL‐C may have influenced outcomes, although adjustment was performed.

Physical activity and dietary intake were assessed with self‐reported questionnaires and may be subject to recall bias or under‐ or over‐reporting. In addition, detailed macronutrient intake could not be quantified. This study is a secondary analysis of the DALI trial and therefore not specifically designed to investigate the role of vitamin D in lipid metabolism, which may introduce a bias. A key strength of this study is the longitudinal assessment of vitamin D and metabolic parameters from early pregnancy through delivery, including cord blood measurements.

## 6 Conclusions

Early vitamin D deficiency before 20 weeks of gestation was associated with altered metabolic trajectories during pregnancy, characterized by greater increases in LDL cholesterol and ketone body concentrations in women with overweight or obesity.

Notably, higher ketone body levels were also observed in cord blood of offsprings of mothers with vitamin D deficiency, suggesting that these metabolic alterations may extend to the fetal environment.

These findings highlight a potential link between early maternal micronutrient status and metabolic adaptations during pregnancy, a critical period for both maternal and fetal health. In particular, our results suggest that vitamin D status may influence dynamic metabolic responses rather than static lipid levels and may be relevant for identifying women at risk of exaggerated metabolic changes.

However, given the observational design, potential residual confounding and inconsistencies in the existing literature, causal relationships cannot be established and clinical recommendations cannot yet be derived.

Previous intervention trials have shown limited effects of vitamin D supplementation on lipid outcomes, indicating that factors such as timing of exposure, baseline vitamin D status and population characteristics may be crucial. Further prospective studies and randomized controlled trials are needed to clarify underlying mechanisms—particularly the role of vitamin D in ketone metabolism—determine whether early or preconception interventions are beneficial, and assess the long‐term implications for maternal and offspring metabolic health.

## Author Contributions

**Jürgen Harreiter:** conceptualization, investigation, writing – original draft, methodology, validation, visualization, writing – review and editing, software, formal analysis, supervision, data curation. **Dorte M. Jensen:** writing – review and editing, investigation, supervision, validation. **Fidelma Dunne:** conceptualization, investigation, methodology, validation, supervision, writing – review and editing. **Teresa Gisinger:** writing – review and editing, visualization, supervision, validation. **Gernot Desoye:** conceptualization, investigation, funding acquisition, methodology, validation, project administration, supervision, writing – review and editing. **Sander Galjaard:** investigation, writing – review and editing, validation, supervision, methodology. **Lilian C. Mendoza:** writing – review and editing, validation, supervision, investigation, data curation. **Roland Devlieger:** conceptualization, investigation, methodology, validation, supervision, writing – review and editing. **David Simmons:** conceptualization, investigation, funding acquisition, methodology, validation, writing – review and editing, project administration, supervision. **Lise Lotte T. Andersen:** investigation, validation, writing – review and editing, supervision. **Ewa Wender‐Ozegowska:** investigation, methodology, validation, writing – review and editing, supervision. **Judith G. M. Jelsma:** supervision, validation, writing – review and editing, investigation, methodology, data curation. **Elisabeth R. Mathiesen:** conceptualization, investigation, methodology, validation, supervision, writing – review and editing. **Mireille N. M. van Poppel:** conceptualization, investigation, funding acquisition, methodology, validation, writing – review and editing, data curation, supervision, resources, project administration. **Annunziata Lapolla:** investigation, validation, writing – review and editing, supervision. **Peter Damm:** conceptualization, investigation, methodology, supervision, writing – review and editing, validation. **David Hill:** conceptualization, investigation, methodology, validation, writing – review and editing, supervision. **Agnieszka Zawiejska:** investigation, validation, writing – review and editing, supervision. **Rosa Corcoy:** conceptualization, investigation, methodology, validation, supervision, writing – review and editing. **Christof Worda:** investigation, writing – review and editing, validation, supervision. **Alexandra Kautzky‐Willer:** conceptualization, investigation, funding acquisition, writing – review and editing, methodology, validation, project administration, supervision, resources. **Frank J. Snoek:** supervision, validation, writing – review and editing, investigation. **Maria G. Dalfra:** investigation, writing – review and editing, supervision, validation. **Dagmar Bancher‐Todesca:** investigation, methodology, validation, writing – review and editing, supervision. **Alessandra Bertolotto:** investigation, validation, writing – review and editing, supervision.

## Funding

DALI has received funding from the European Community's 7th Framework Programme (FP7/2007–2013) under grant agreement no. 242187. In the Netherlands, additional funding was provided by the Netherlands Organization for Health Research and Development (ZonMw) (grant no. 200310013). In Poland, additional funding was obtained from the Polish Ministry of Science (grant no. 2203/7. PR/2011/2). In Denmark, additional funding was provided by the Odense University Free Research Fund. In the United Kingdom, the local DALI team was supported by NIHR Clinical Research Network: Eastern. In Spain, additional funding was provided by CAIBER 1527‐B‐226 and from the Spanish Diabetes Society (SED) XI Grant for clinical research projects in diabetes led by young investigators, awarded to LM in 2020. In Austria, additional funding was provided by the Medical Scientific Fund of the Mayor of Vienna, Grant/Award Number: 23026, 15205. The funders had no role in any aspect of the study beyond funding.

## Ethics Statement

The study was conducted in accordance with the Declaration of Helsinki, and approved by the Institutional Review Board or Ethics Committees of the participating centres: NRES Committee East of England‐Norfolk: 11/EE/0221; Medical University Poznan: 1165/12; UZ KU Leuven: ML7625; Hospital De La Santa Creu i Sant Pau Barcelona 13/006 (OBS); Medical University Vienna: 2022/2012–1369/2013 (date 08.01.2013); Province of Padua and Pisa: 4201 Å~11; Galway University Hospitals: 7/12; Ethical comittee VU medisch centrum Amsterdam: nr. 2012/400; Copenhague and Odense: Scientific Ethics Committee for the Capital Region, Hillerod, Denmark, Protokol nr.: H‐4‐2013‐005.

## Consent

Informed consent was obtained from all subjects involved in the study.

## Conflicts of Interest

The authors declare no conflicts of interest.

## Data Availability

The data that support the findings of this study are available from the corresponding author (J.H.) upon reasonable request.
